# Introduction of new oral antiplatelet drugs in myocardial infarction hospital network: initial experience

**DOI:** 10.1007/s11239-013-0930-z

**Published:** 2013-04-30

**Authors:** Tomasz Rakowski, Artur Dziewierz, Zbigniew Siudak, Paweł Kleczyński, Jacek S. Dubiel, Dariusz Dudek

**Affiliations:** 1 Second Department of Cardiology, Jagiellonian University Medical College, Kopernika 17 Street, 31-501 Krakow, Poland; 2 Department of Interventional Cardiology, Jagiellonian University Medical College, Kopernika 17 Street, 31-501 Krakow, Poland

## To the editor

New oral antiplatelet drugs (prasugrel, ticagrelor) are recommended in the current European Society of Cardiology guidelines for the management of patients presenting with ST-segment elevation myocardial infarction (STEMI) [1]. However, in many STEMI hospital networks administration of a 600-mg clopidogrel loading dose before or during transfer to cathlab (including in ambulance administration) is a standard of care, since this strategy has been implemented for many years. Early administration of the drug may enhance antiplatelet effects of clopidogrel at the time of primary percutaneous coronary intervention (PCI), in comparison to the administration in the cathlab. On the other hand, the response to clopidogrel in patients with STEMI, especially in patients with hemodynamic compromise is impaired. Prasugrel and ticagrelor are more potent antiplatelet drugs, with faster and more profound antiplatelet effect. These agents are preferred over clopidogrel, if not contraindicated, in patients with STEMI since both are superior in comparison to clopidogrel in terms of the reduction of ischemic events [2, 3]. However, introduction of prasugrel and ticagrelor may need to change STEMI network logistics since those new drugs are predominantly administered in the cathlab, but not in ambulances or in non-PCI centers before transportation [4].

In our high-volume primary PCI center, early (before transfer to cathlab) administration of acetylsalicylic acid, unfractionated heparin and a 600-mg clopidogrel loading dose has been a well-established standard of treatment from many years. In-cathlab administration of antiplatelet drugs was a rare strategy so it was necessary to reorganize STEMI network for new antiplatelet drugs introduction in daily practice. An observational, prospective registry was designed to describe the implementation of new oral antiplatelet drugs in our network. First 100 consecutive STEMI patients (no exclusion criteria) admitted to our center after introduction of prasugrel and ticagrelor were enrolled. Registry was focused on antiplatelet therapy including type of drug, moment of administration, time from administration to PCI. Data on reason for the administration of clopidogrel instead of new drugs was also collected. Additionally, platelet aggregation inhibition was assessed at the time of PCI (guide wire introduction) with Plateletworks^®^ Aggregation Kits (Helena Laboratories, Beaumont, TX, USA) [5]. The registry analyzed the current clinical practice and did not modify patients diagnostics and treatment.

**Fig. 1:**
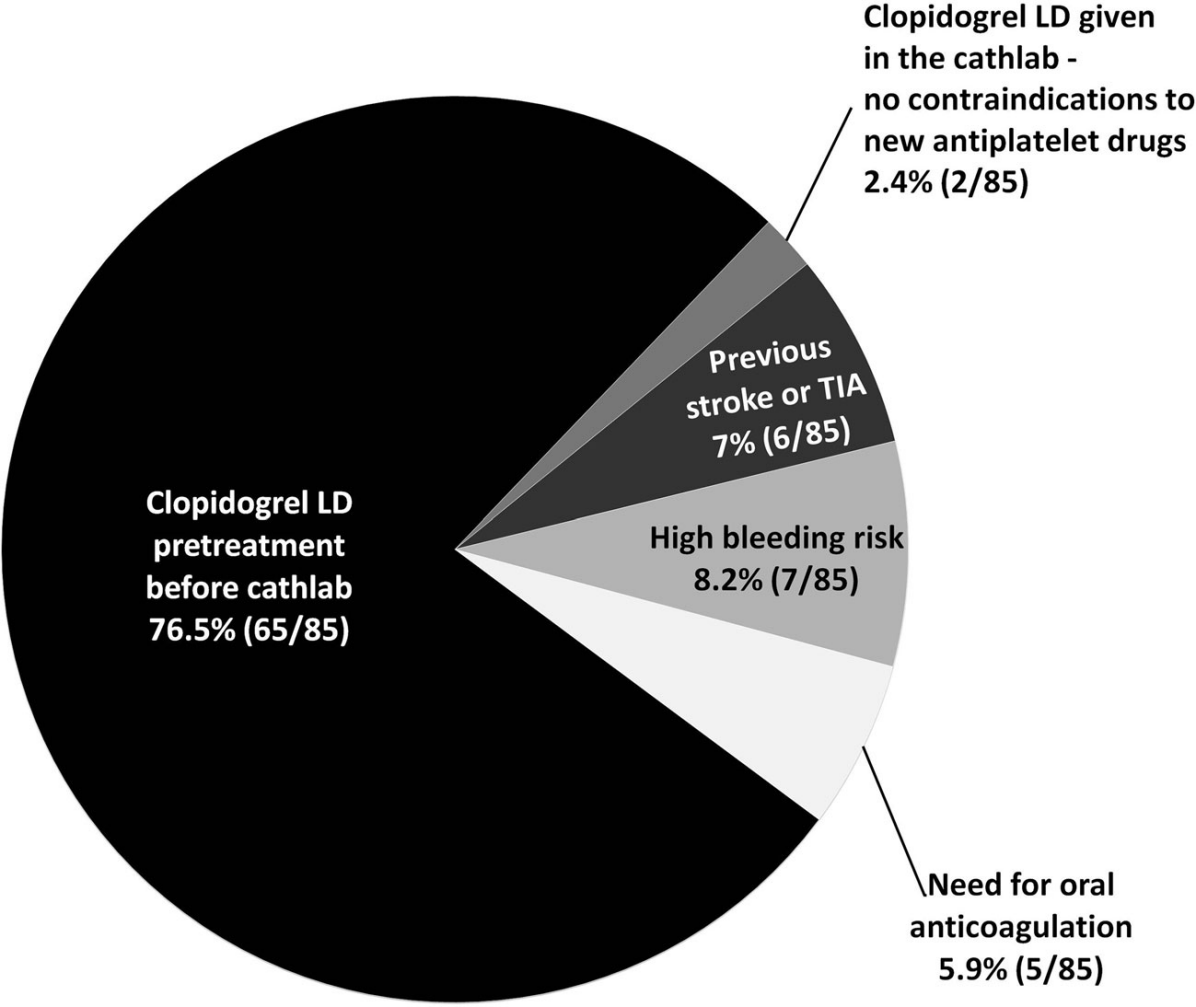
Reasons for the treatment with clopidogrel instead of new antiplatelet drugs (prasugrel/ticagrelor). *LD* loading dose, *TIA* transient ischemic attack

A total of 100 consecutive STEMI patients entered the registry. Clinical characteristics of patient population are presented in Table 1. Registry represents real life STEMI population including elderly patients and patients in cardiogenic shock. Acetylsalicylic acid was administered before transfer to cathlab in all patients. New oral antiplatelet drugs were given after cathlab admission before or during coronary angiography only in 15 out of 100 patients (13 patients treated with a 60-mg prasugrel loading dose; 2 patients treated with a 180-mg ticagrelor loading dose). In the remaining 85 patients a 600-mg clopidogrel loading dose was given before (80 patients) or after admission to the cathlab (5 patients). Reasons for the administration of clopidogrel instead of new antiplatelet drugs are presented in Fig. 1. The contraindications for prasugrel/ticagrelor were present in 18 patients. In 65 patients the main cause of new therapy introduction failure was clopidogrel loading dose given before transfer to the cathlab (according to earlier everyday practice). The time from the administration of loading dose to PCI was significantly longer in patients treated with clopidogrel than with new antiplatelet drugs (median [IQR]: 75 [60–110] vs 15 [12.5–20] min; *p* < 0.0001). There was no significant difference in the platelet aggregation inhibition at the time of PCI between groups. However, a trend towards higher inhibition in clopidogrel pretreated patients was observed (median [IQR]: 55 % [43–63] vs 48 % [41–51]; *p* = 0.08). PCI was performed in 97 % of patients with aspiration thrombectomy in more than half of patients, and with the wide range of stents types used (Table 1). Abciximab was administered during PCI in 28 patients (in three patients treated with prasugrel) after platelet function assessment.

**Table 1:** Baseline characteristics, concomitant medications and interventional treatment details

Variable	STEMI patients (*n* = 100)
Males	69/100
Age [years]	62 [56–73]
Age ≥75 years	23/100
Age ≥80 years	14/100
Diabetes mellitus	29/100
Arterial hypertension	50/100
Previous stroke/TIA	6/100
Previous myocardial infarction	9/100
Previous PCI	9/100
Cardiogenic shock on admission	10/100
Anterior wall infarction	43/100
Stent thrombosis	0/100
PCI	97/100
TIMI 3 flow grade after PCI	93/100
Aspiration thromectomy	52/97
Stent	90/97
DES	45/90
Mesh-covered (MGuard) stent	14/90
Self-expandable (Stentys) stent	4/90
BMS (non MGuard, non Stentys)	27/90

Values are presented as number of patients or medians [inter-quartile range]

*BMS* bare metal stent, *DES* drug eluting stent, *PCI* percutaneous coronary intervention, *TIA* transient ischemic attack, *TIMI* thrombolysis in myocardial infarction

Our data showed that introduction of new antiplatelet drugs may be slowed down by well established strategy of early clopidogrel loading dose administration. In such circumstances, the administration of prasugrel/ticagrelor on top of clopidogrel may be an option since all those drugs competitively block one receptor (known main mode of action) but clinical data on the safety of such approach are relatively limited. PLATO study has included patients pretreated with clopidogrel and ticagrelor administration is recommended by current STEMI Guidelines also in clopidogrel pretreated patients [1, 2]. However, the assessment of ticagrelor administration on top of clopidogrel was not the primary objective of PLATO study. The clinical evidence of prasugrel administration in clopidogrel pretreated patients is weaker and it is based on platelet reactivity studies (not powered for safety) [3, 6, 7]. Nevertheless, the administration of new antiplatelet drugs in clopidogrel pretreated is gaining popularity in clinical practice. The time from initial STEMI diagnosis to primary PCI is still important problem of reperfusion treatment strategy in real-life. This also causes the delay from STEMI diagnosis to new antiplatelet drugs administration in the cathlab. In our registry clopidogrel was administered more than 1 h earlier than prasugrel/ticagrelor what may influence the platelet inhibition at the time of PCI despite more rapid action of new antiplatelet drugs. Moreover, the response to antiplatelet drugs in patients with STEMI may be delayed not only for clopidogrel but also for new oral antiplatelet drugs. However, there is no data on comparison of those two strategies (early clopidogrel vs in cathlab prasugrel/ticagrelor loading dose) in large scale clinical trials. In the networks with established strategy of early clopidogrel administration, switch for early new oral antiplatelet drugs may be an option but there is no clinical data on safety and efficacy of such approach (the ATLANTIC (STEMI) and ACCOAST (NSTEMI) trials are ongoing). That strategy of early platelet inhibition has been described with abciximab and showed benefit in high-risk patients [8, 9]. On the other hand, in-cathlab drugs administration may play important role when urgent surgery is required but this is a very rare scenario in STEMI patients. In some STEMI patients prasugrel and/or ticagrelor are contraindicated. For those patients early risk stratification should be performed just after STEMI diagnosis since clopidogrel administration before transfer may be a valuable option if new drugs are contraindicated. Our data has important limitation including: 1/low number of patients, but we have focused on first group of patients after new drug introduction to define logistics problems and upgrade the implementation process (currently the penetration of prasugrel/ticagrelor is about 30 %); 2/registry design which reduces the value of platelet function analysis, but the study was conducted to observe real-life clinical practice.

In conclusion, initiation of new oral antiplatelet drugs in STEMI networks needs to change the treatment logistics since early clopidogrel administration is established strategy for most of STEMI patients in daily practice. Tailored antiplatelet therapy approach is necessary since as of today three oral P2Y12 antagonists with different contraindications are available. Despite results of large scale clinical trials there are still some gaps in evidence concerning new antiplatelet drugs usage in real-life STEMI patients in daily practice. This includes: usage of new antiplatelet drugs on top of clopidogrel loading dose; optimal antiplatelet treatment choice for early administration when transfer for primary PCI is necessary; optimal antiplatelet treatment strategy for patients after stroke/TIA, patients with need for oral anticoagulation.
